# Anatomical, physiological, and logistical indications for the open abdomen: a proposal for a new classification system

**DOI:** 10.1186/s13017-016-0083-4

**Published:** 2016-06-14

**Authors:** Joao Rezende-Neto, Timothy Rice, Emanuelle Savio Abreu, Ori Rotstein, Sandro Rizoli

**Affiliations:** Department of Surgery Division of General Surgery, University of Toronto, St. Michael’s Hospital, 30 Bond Street, Rm 3-074 Donnelly Wing, Toronto, ON M5B 1 W8 Canada; Hospital Risoleta Tolentino Neves, Federal University of Minas Gerais, Minas Gerais, Brazil

**Keywords:** Open abdomen, Trauma, Acute care surgery, Classification

## Abstract

**Background:**

A systematic approach to the appropriate use of the open abdomen strategy has not been described. We propose three fundamental reasons for the use of this strategy, anatomical, physiological and logistical. Anatomical reasons pertain to the inability to bring the fascial edges together including soft tissue defects. Physiological reasons relate to features of systemic dysfunction. Logistical reasons involve any anticipated abdominal re-intervention while preserving fascia. These categories occur either as a single reason or in any combination.

**Methods:**

A single-center prospective observational study of patients with open abdomens in trauma and acute abdomen. Surgeons were asked to select from the three reasons (single or any combination of) their motivation for using the open abdomen upon completion of the initial operation. Patients were compared using the non-parametric Wilcoxon two-sample test or Kruskal-Wallis test. Chi-square or Fisher’s exact test was used for categorical variables; Statistical significance set at *P*-value ≤ 0.05.

**Results:**

Forty-five consecutive patients with open abdomens were evaluated (Jan. 1- Dec. 31, 2012). Mean age was 38.8 years, 32 were male, 39 (86.7 %) sustained trauma. The most common single reason for the open abdomen was physiological (24.4 %), 33 patients had multiple reasons, the most common combination being anatomical and physiological (22.2 %). A physiological reason was linked to: lower pH, higher lactate, and lower systolic blood pressure on admission (*p* < 0.05). A logistical reason was associated with earlier primary fascial closure, intra-operative packing, and bowel left in discontinuity. Logistic regression and adjusted odds ratio of primary fascial closure was: physiological (0.08, 95 % CI, 0.01–0.92, *p* = 0.043); logistical (6.03, 95 % CI, 1.13–32.29, *p* = 0.036); and anatomical (0.83, 95 % CI, 0.16–4.18, *p* = 0.816).

**Conclusion:**

We defined three basic indications for the use of the open abdomen, anatomical physiological and logistical. These indications establish a practical and comprehensive terminology that could help to promote appropriate use of the open abdomen.

## Background

The open abdomen is considered a hallmark of damage control surgery. With the widespread use of this staged-approach to the laparotomy, the open abdomen has become an acceptable option in the operative management of critically ill patients [1, 2]. Despite the use of the open abdomen technique by trauma and acute care surgeons, the fundamental indications that define its appropriate application are poorly understood [3–5]. The need to establish consensus indications was made clear by a recent meta-analysis reporting over one thousand indications for damage control surgery found throughout the literature [6]. The indications for the open abdomen and damage control surgery are often applied interchangeably. In many instances however, the open abdomen strategy can be used outside the context of the physiologic abnormalities associated with damage control surgery. Thus, the current indications for damage control surgery may not be sufficiently broad to guide the use of the open abdomen [3, 7]. Consequently, a comprehensive definition of the indications for the appropriate use of the open abdomen is required [8].

The purpose of this study was to introduce a classification system supported by clinical data that provides practical and comprehensive terminology of the indications for the open abdomen.

## Methods

The Research Ethics Committee of the Risoleta Tolentino Neves Hospital approved the conduct of this study (resolution number 196/96/23/2/07), informed consent was waived. This was a single-center prospective observational study.

Patients were screened on weekdays between 7:00–19:00 h at the Hospital Universitario Risoleta Tolentino Neves, a regional trauma center affiliated with the Federal University of Minas Gerais, located in Belo Horizonte, Brazil. This time interval relates to availability of research assistants at the institution. Patients admitted to the trauma acute care surgery service that required a laparotomy for trauma or an acute abdomen where an open abdomen strategy was used fulfilled the study inclusion criteria. Patients were excluded if they were less than 18 years of age.

Research assistants approached the primary surgeon in the operating room after the decision to use an open abdomen technique was already made. The acute care surgery/trauma call schedule of the institution has a different team of surgeons on call each day of the week working on 12 h shifts. All surgeons who performed the operations were experienced general surgeons with subspecialization in trauma and acute care surgery. These surgeons were given a questionnaire that described the nature of the study and were asked to select one or more reasons for why they decided to leave the abdomen open. Management of the patients remained entirely at the discretion of the surgeons.

We defined three basic indications for the use of the open abdomen, anatomical physiological and logistical:

- Anatomical indications are represented by the inability to approximate the edges of the laparotomy and perform primary closure, soft tissue loss, or impending risk of abdominal compartment syndrome.
- Physiological indications pertain to a severe physiologic derangement of the patient requiring damage-control damage control strategies.
- Logistical indications occur when serial surgical interventions are necessary while preserving fascia.

Surgeons were also allowed not to choose any of the aforementioned indications and/or add additional reasons for the open abdomen. All questionnaires were completed before the surgeon left the operating room and were given directly back to the research coordinator. Research personnel assessed patients daily for clinical outcomes. Patients were followed until the time of hospital discharge or when censored at 7 days.

### Statistical analysis

Descriptive statistics were calculated as means, standard deviations, medians, interquartile ranges, and proportions. Patient groups were compared using the non-parametric Wilcoxon two-sample test or Kruskal-Wallis test for age, hospital length of stay, Intensive care Unit (ICU) length of stay, and Injury Severity Score (ISS). Chi-square or Fisher’s exact test was used for categorical variables. The association between all three reasons for abdominal closure failure and death was estimated from a logistic regression model (odds ratios and 95 % confidence intervals). All analyses were performed using SAS 9.4 (SAS Institute, Inc., Cary, NC) and SPSS Statistics 22 (IBM, Inc.). Statistical significance was set at a two-sided *P*-value of 0.05 or less.

## Results

**Fig. 1:**
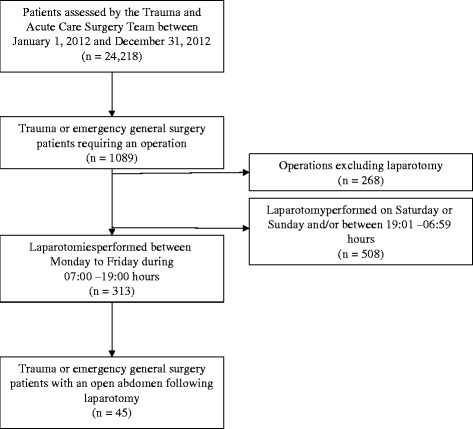
Consort flow chart describing patients enrolled in the study. Patients assessed by the trauma and acute care surgery team include all patients that required emergency surgical consultation and traumas of all severities

A total of 24,218 patients were assessed by the trauma acute care surgery service from January 1, 2012 to December 31, 2012. This included patients presenting with all types of surgical pathologies and traumas of all magnitudes, including minor injuries. Of these, 821 patients required a laparotomy and 313 were screened for eligibility. Another 508 patients also had a laparotomy but were not screened because the operations were not performed during the study hours; 64 of those patients had an open abdomen (12.6 %). Enrollment is outlined in the CONSORT diagram in Fig. 1.

Forty-five patients had an open abdomen following a laparotomy for either trauma or acute abdomen, on weekdays between 7:00 and 19:00 h, and were enrolled in the study.

The baseline characteristics of the cohort according to each of the three reasons are presented in Table 1.

**Table 1:** Baseline characteristics and laboratory values among physiological, anatomical, and logistical reasons in patients with open abdomens

	Physiological (*n* = 45)			Anatomical (*n* = 45)			Logistical (*n* = 45)		
	Present (*n* = 38)	Absent (*n* = 7)	*p*	Present (*n* = 25)	Absent (*n* = 20)	*p*	Present (*n* = 21)	Absent (*n* = 24)	*p*
Age, mean (SD),yrs	37.9 (18.0)	43.7 (23.6)	0.800	40.8 (19.2)	36.4 (18.4)	0.299	40.4 (22.1)	37.6 (15.7)	0.731
Male, n (%)	28 (73.7)	4 (57.1)	0.394	16 (64.0)	16 (80.0)	0.327	15 (71.4)	17 (70.8)	1.000
Trauma mechanism, n (%)									
Penetrating	20 (52.6)	3 (42.9)	0.699	11 (44.0)	12 (60.0)	0.373	9 (42.9)	14 (58.3)	0.376
Blunt	13 (34.2)	3 (42.9)	0.686	11 (44.0)	5 (25.0)	0.224	7 (33.3)	9 (37.5)	1.000
Acute Abdomen, n (%)	5 (13.2)	1 (14.3)	1.000	3 (12.0)	3 (15.0)	1.000	5 (23.8)	1 (4.2)	0.083
Other reasons present, n (%)									
Logistical	17 (44.7)	4 (57.1)	0.422	12 (48.0)	9 (45.0)	1.000	-	-	-
Anatomical	19 (50.0)	6 (85.7)	0.112	-	-	-	12 (57.1)	13 (54.2)	1.000
Physiological	-	-	-	19 (76.0)	19 (95.0)	0.112	17 (81.0)	21 (87.5)	0.689
ISS, mean (SD)	22.7 (5.5)	19.0 (3.2)	0.143	21.2 (5.0)	23.4 (5.6)	0.193	22.8 (4.8)	21.7 (5.7)	0.389
T < 36, n (%)	21 (55.3)	1 (14.3)	0.096	11 (44.0)	11 (55.0)	0.554	7 (33.3)	15 (62.5)	0.075
T > 38, n (%)	4 (10.5)	1 (14.3)	1.000	3 (12.0)	2 (10.0)	1.000	3 (14.3)	2 (8.3)	0.652
pH < 7.2, n (%)	27 (71.1)	0	0.001	15 (60.0)	12 (60.0)	1.000	11 (52.4)	16 (66.7)	0.374
Lactate > 5, n (%)	24 (63.2)	1 (14.3)	0.034	13 (52.0)	12 (60.0)	0.764	11 (52.4)	14 (58.3)	0.769
WBC < 4, n (%)	2 (5.3)	0	1.000	0	2 (20.0)	0.192	0	2 (8.3)	0.491
WBC > 12, n (%)	14 (36.8)	4 (57.1)	0.412	9 (36.0)	9 (45.0)	0.559	13 (61.9)	5 (20.8)	0.007
Mechanical ventilation, n (%)	38 (100)	7 (100 %)	-	25 (100)	20 (100)	-	21 (100)	24 (100)	-
Fi0_2_ > 40 %, n (%)	29 (76.3)	3 (42.9)	0.168	17 (68.0)	15 (75.0)	0.745	16 (76.2)	16 (66.7)	0.528
Coagulopathy, n (%)	30 (78.9)	4 (57.1)	0.337	16 (64.0)	18 (90.0)	0.079	16 (76.2)	18 (75.0)	1.000
SBP < 90, n (%)	35 (92.1)	4 (57.1)	0.039	22 (88.0)	17 (85.0)	1.000	18 (85.7)	21 (87.5)	1.000
AKI, n (%)	4 (10.5)	0	1.000	2 (8.0)	2 (20.0)	1.000	4 (19.0)	0	0.040
Hemodialysis, n (%)	1 (2.6)	0	1.000	1 (4.0)	0	1.000	1 (4.8)	0	0.467
ACS, n (%)	1 (2.6)	1 (14.3)	0.290	2 (80)	0	0.495	0	2 (8.3)	0.491

*SD* standard deviation, *ISS* injury severity score, *T* temperature, *WBC* white blood cell count, *Fi0*_*2*_ fraction of inspired oxygen, *SBP* systolic blood pressure, *AKI* acute kidney injury, *ACS* abdominal compartment syndrome

The mean age was 38.8 ± 18.8 years old and 32 (71.1 %) patients were male. Thirty-nine (86.7 %) patients required a laparotomy for trauma. The mean Injury Severity Score was 22.2 (interquartile range, 16–34). Temporary abdominal closure was performed using a Bogota bag in 40 (88.9 %) patients and negative pressure wound therapy in 3 (6.7 %) patients. Both temporary abdominal closure techniques were used in 2 (4.4 %) patients.

Patients with physiological reasons compared to those without physiological reasons had a significantly higher lactate (lactate > 5.0 mmol/L: 63.2 % vs. 14.3 %; *p* = 0.034); lower pH (pH < 7.2: 71.1 % vs. 0 %; *p* < 0.001) and lower systolic blood pressure (SBP < 90 mmHg: 92.1 % vs. 57.1 %; *p* = 0.039) on admission.

Fifteen (33.3 %) patients had a single reason for leaving the abdomen open. The most common single reason for the open abdomen was physiologic (*n* = 11, 24.4 %). Of the 30 patients with multiple reasons, 27 (90 %) had a physiologic reason. Physiologic and anatomic reasons were the most frequently identified combinations (*n* = 10, 22.2 %).

Primary fascial closure was obtained in 19 (42.2 %) patients (Table 2).

**Table 2:** Outcomes among physiological, anatomical, and logistical reasons in patients with open abdomens

	Physiological (*n* = 45)			Anatomical (*n* = 45)			Logistical (*n* = 45)		
	Present (*n* = 38)	Absent (*n* = 7)	*p*	Present (*n* = 25)	Absent (*n* = 20)	*p*	Present (*n* = 21)	Absent (*n* = 24)	*p*
ICU LOS (IQR)	5 (1–5)	15 (12–21)	0.230	12 (1–27.5)	4.5 (1–11.8)	0.399	10 (1.5–24)	4.5 (1–27.8)	0.541
Hospital LOS (IQR)	7 (1–7)	33 (22–43)	0.133	27.8 (1–41)	7 (1–21.5)	0.495	19 (2.5–42.5)	5 (1–34.5)	0.300
Primary fascial cl, n (%)	13 (34.2)	6 (85.7)	0.031	11 (44.0)	8 (40.0)	1.000	13 (61.9)	6 (25.0)	0.017
0–24 h	2 (5.3)	1 (14.3)	0.405	0	3 (15.0)	0.080	3 (14.3)	0	0.094
24–48 h	1 (2.6)	3 (42.9)	0.009	3 (12.0)	1 (5.0)	0.617	2 (9.5)	2 (8.3)	1.000
48–72 h	3 (7.9)	1 (14.3)	0.505	2 (8.0)	2 (10.0)	1.000	3 (14.3)	1 (4.2)	0.326
72–96 h	2 (5.3)	0	1.000	2 (8.0)	0	0.495	1 (4.8)	1 (4.2)	1.000
96–120 h	1 (2.6)	1 (14.3)	0.290	2 (8.0)	0	0.495	2 (9.5)	0	0.212
120–144 h	1 (2.6)	0	1.000	1 (4.0)	0	1.000	1 (4.8)	0	0.467
144–168 h	3 (7.9)	0	1.000	1 (4.0)	2 (10.0)	0.577	1 (4.8)	2 (8.3)	1.000
Fascial cl ≤ 72 h, n (%)^a^	6 (15.8)	5 (71.4)	0.004	5 (20.0)	6 (30.0)	0.500	8 (38.1)	3 (12.5)	0.048
Mortality, n (%)	19 (50.0)	1 (14.3)	0.112	10 (40.0)	10 (50.0)	0.557	7 (33.3)	13 (54.2)	0.231
0–24 h	13 (34.2)	1 (14.3)	0.407	8 (32.0)	6 (30.0)	1.000	5 (23.8)	9 (37.5)	0.356
24–48 h	1 (2.6)	0	1.000	0	1 (5.0)	0.444	0	1 (4.2)	1.000
48–72 h	2 (5.3)	0	1.000	0	2 (10.0)	0.192	0	2 (8.3)	0.491
72–96 h	2 (5.3)	0	1.000	1 (4.0)	1 (5.0)	1.000	1 (4.8)	1 (4.2)	1.000
96–120 h	1 (2.6)	0	1.000	1 (4.0)	0	1.000	1 (4.8)	0	0.467
120–144 h	0	0	-	0	0	-	0	0	-
144–168 h	0	0	-	0	0	-	0	0	-
Cause of death, n (%)									
Sepsis	4 (10.5)	1 (14.3)	1.000	3 (12.0)	2 (10.0)	1.000	2 (9.5)	3 (12.5)	1.000
MOF	10 (26.3)	1 (14.3)	0.663	3 (12.0)	8 (40.0)	0.041	2 (9.5)	9 (37.5)	0.040
Bleeding	12 (31.6)	1 (14.3)	0.654	9 (36.0)	4 (20.0)	0.327	6 (28.6)	7 (29.2)	1.000

*ICU* intensive care unit, *LOS* length of stay, *IQR* interquartile range, *cl* closure, *MOF* multi-organ failure

^a^statistically significant difference compared to fascia closed >72 h and fascia not closed

Eleven patients (58 %) had primary fascial closure within 72-h. Rates of primary fascial closure within 72-h was highest among those with logistical reasons (38.1 %) compared to 12.5 % if a logistical reason was absent (*p* = 0.048). Patients with physiological reasons were less likely to have primary fascial closure compared to those without physiological reasons (34.2 % vs. 85.7 %, OR, 0.09; 95 % CI, 0.01–0.80; *p* = 0.031). Patients with logistical reasons were more likely to have primary fascial closure during hospital stay than those without logistical reasons (61.9 % vs. 25.0 %, OR, 4.88; 95 % CI, 1.36–17.47; *p* = 0.012). There was no significant association between primary fascial closure among patients with anatomical reasons or among those with multiple reasons compared to single reasons (*p* = 0.787 and *p* = 0.393, respectively).

Because a physiological reason was highly prevalent, we also performed a logistic regression analysis to examine the joint association of all three reasons with successful primary fascial closure. In logistic regression, the adjusted odds ratio (AOR) of primary fascial closure was as follows: physiological (0.08, 95 % CI, 0.01–0.92; *p* = 0.043); logistical (6.03, 95 % CI, 1.13–32.29; *p* = 0.036); and anatomical (0.83, 95 % CI, 0.16–4.18; *p* = 0.816) (Table 3).

**Table 3:** Logistic regression model results of variables related to primary fascial closure

Factors	OR	95 % Wald confidence intervals	*p*
Age	1.06	0.99–1.13	0.1050
Logistical	6.03	1.13–32.29	0.0358
Anatomical	0.83	0.16–4.18	0.8158
Physiological	0.08	0.01–0.92	0.0425
Blunt mechanism	0.20	0.02–1.75	0.1476
Acute Abdomen	0.43	0.01–16.14	0.6456

*OR* odds ratio

Intraoperatively, patients with logistical reasons had significantly higher rates of packing than when that reason was not present (42.9 % vs. 0 % *p* < 0.001). Bowel resections and bowel left in discontinuity were also more common in patients with logistical reasons; respectively (57.1 % vs. 12.5 %; *p* = 0.004 and 42.9 % vs. 0 %; *p* < 0.001) (Table 4).

**Table 4:** Temporary abdominal closure strategy and operative interventions among physiological, anatomical, and logistical reasons in patients with open abdomens

	Physiological (*n* = 45)			Anatomical (*n* = 45)			Logistical (*n* = 45)		
	Present (*n* = 38)	Absent (*n* = 7)	*p*	Present (*n* = 25)	Absent (*n* = 20)	*p*	Present (*n* = 21)	Absent (*n* = 24)	*p*
TAC strategy, n (%)									
NPWT	5 (13.2)	0	0.577	2 (8.0)	3 (15.0)	0.642	4 (19.0)	1 (4.2)	0.169
Bogota bag	35 (92.1)	7 (100)	1.000	23 (92.0)	19 (95.0)	1.000	19 (90.5)	23 (95.8)	0.592
Intraoperative procedures, n (%)									
Primary repair of hollow viscus	10 (26.3)	0	0.320	3 (12.0)	7 (35.0)	0.083	3 (14.3)	7 (29.2)	0.296
GI tract in discontinuity	7 (18.4)	2 (28.6)	0.614	5 (20.0)	4 (20.0)	1.000	9 (42.9)	0	<0.001
Bowel resection	13 (34.2)	2 (28.6)	1.000	9 (36.0)	6 (30.0)	0.757	12 (57.1)	3 (12.5)	0.004
Packing	7 (18.4)	2 (28.6)	0.614	5 (20.0)	4 (20.0)	1.000	9 (42.9)	0	<0.001
Splenectomy	9 (23.7)	1 (14.3)	1.000	6 (24.0)	4 (20.0)	1.000	4 (19.0)	6 (25.0)	0.729
Repair of major vessel	5 (13.2)	1 (14.3)	1.000	3 (12.0)	3 (15.0)	1.000	4 (19.0)	2 (8.3)	0.396
Ligation of major vessel	4 (10.5)	1 (14.3)	1.000	4 (16.0)	1 (5.0)	0.362	3 (14.3)	2 (8.3)	0.689
Drainage of IAA	1 (2.6)	1 (14.3)	0.290	2 (8.0)	0	0.495	1 (4.8)	1 (4.2)	0.652
Nephrectomy	2 (5.3)	1 (14.3)	0.405	3 (12.0)	0	0.242	1 (4.8)	2 (8.3)	1.000
Repair of solid organ	9 (23.7)	0	0.315	4 (16.0)	5 (25.0)	0.482	3 (14.3)	6 (25.0)	1.000
Ostomy	5 (13.2)	0	0.577	4 (16.0)	1 (5.0)	0.362	3 (14.3)	2 (8.3)	0.652

*TAC* temporary abdominal closure, *NPWT* negative pressure wound therapy, *GI* gastrointestinal tract, *IAA* intra-abdominal abscess

The overall mortality rate was 44.4 %. Of the 20 patients that died, 14 (70 %) died within the first 24-h. Mortality was most common in patients with physiological reasons (*n* = 19, 50.0 %); however, none of the reasons were significantly associated with mortality. Bleeding was the most common cause of death for all reasons. Multi-organ failure as a cause of death was lower in patients with anatomical (anatomical 12.0 % vs. without anatomical 40 %; *p* = 0.041) and logistical reasons (logistical 9.5 % vs. 37.5 % without logistical reason; *p* = 0.040).

## Discussion

The open abdomen strategy is commonly used in modern surgical practice. With greater understanding of damage control principles it has become widely adopted by trauma and acute care surgeons [1]. Our knowledge of the open abdomen has, however, trailed behind our enthusiasm, as the indications guiding its appropriate use remain undefined. Furthermore, despite employing techniques previously described to repair incisional ventral hernias and the significant improvement in mesh construction early closure of the open abdomen remains a challenge [9, 10]. The average rate of primary fascial closure was 62 % in a recent systematic review and meta-analysis involving more than 3000 patients with open abdomens [11]. It was demonstrated in that study, after adjusting for patient heterogeneity, that primary fascial closure had a significant role in mortality reduction, decreasing complications and hospital length of stay [11]. These findings highlight the importance of judicious use of the open abdomen strategy.

Progress towards better defining its indications has been hampered by the lack of a common language and the absence of the widespread adoption of an open abdomen classification system. With the introduction of consensus definitions and a new system of standardized nomenclature, there has been some headway with the former but a robust classification system is still missing [5, 12]. Additionally, diverse practice patterns between studies and the inclusion of heterogeneous population of patients have contributed to the paucity of evidence-based data [3, 6]. The need for higher quality data in all facets of the management of the open abdomen has been the impetus for the development of an International Register of Open Abdomen promoted by the World Society of Emergency Surgery [3]. Nevertheless, no level one recommendations can be made with respect to the indications for the open abdomen in damage control or emergency general surgery [3, 7].

The classification presented herein, defines three categories that encompass all of the indications for the open abdomen: anatomical; physiological; and logistical. We believe that this classification has practical application and effectively homogenizes patients based on clinical features and outcomes. Furthermore, it is applicable to both trauma and non-trauma populations, and introduces a simple standardized nomenclature that will facilitate communication and future studies. Moreover, our classification in no way negates previously defined indications for the open abdomen in damage control surgery [13–18].

Damage control surgery laid the foundation for our understanding of the open abdomen and, as a result, the use of the open abdomen in modern surgical practice has largely been extrapolated from the damage control surgery literature. Hence, it is not surprising that the most common category used to define the indications for the open abdomen is based on patient’s physiologic parameters [13, 19]. This current framework is overly restrictive and neglects other important considerations relevant to the open abdomen. Dichotomizing the open abdomen and damage control surgery is important to successfully define its appropriate use.

A classification system derived from local findings specific to the open abdomen was originally proposed by Bjork et al [5, 20]. This classification describes four categories that are based on the degree of adhesions and degree of enteric contamination. One of the strengths of this classification lies in its ability to describe the natural history and grade the progressively increasing complexity of the open abdomen management [21]. Included in the most complex category are, enteric leak, deteriorating grade, and the presence of an enteroatmospheric fistula. All have been shown to reliably predict a worse clinical outcome [20, 21]. This classification, however, has limited usefulness in its ability to establish the initial indications for leaving the abdomen open [22]. Arguably, knowledge of the initial indication should be an integral component of a classification system and is a requisite for facilitating communication between clinicians and investigators. Moreover, since the determinants of the aforementioned classification are predominantly anatomically based it is not sufficiently broad to encompass all of the potential contexts where an open abdomen strategy may be applied.

Since surgeons in our study reported their reasons for leaving the abdomen open immediately after the conclusion of the operation, our findings provide an accurate representation of current surgical practice and offer insight into surgeons’ motives for using this surgical strategy. Physiological reasons were the most prevalent indication for the open abdomen in our study. This finding is consistent with a survey of American Association for the Surgery of Trauma members in which respondents reported, “damage control surgery” (a surrogate for physiological derangements) as the most common reason for leaving the abdomen open. This was followed by anatomical reasons: “abdominal organ distention” and “inability to close fascia”; and then logistical reasons: “preparation for a 2nd look” [1]. We also observed a similar distribution in the frequency in our study with anatomical reasons being more common than logistical. However, our findings are at variance with the results of a 2004 survey of Canadian Trauma Surgeons, in which 87 % of respondents reported an anatomical reason, “unbridgeable gap”, followed by a logistical reason, “planned re-operation”, as being the two most frequently reported indications for the open abdomen [2]. The findings of this survey may simply reflect the evolution of our understanding of the open abdomen over a 10-year period but may also reveal the significant variability in practice patterns that exist [23].

In our study, patients with physiological reasons for the open abdomen were also less likely to have primary fascial closure compared to those without physiological reasons. The higher admission lactate level, lower pH, and lower systolic blood pressure in those with physiological reasons lends further support to the association between the severity of physiologic derangement and the subsequent inability to obtain primary fascial closure. This link was reported in a prospective multi-institutional study that identified risk factors for failure to achieve primary fascial closure among trauma patients with an open abdomen [24]. The authors similarly found that patients who did not have primary fascial closure were more likely to have a lower admission pH and a lactate ≥ 7.5 [24]. Interestingly, the presence of an additional reason (anatomical and/or logistical) to a physiological reason did not confer an increased risk of failure to obtain primary fascial closure in our series. The eleven patients who had isolated physiological reason may therefore represent a cohort of patients at greatest risk for failed fascial closure. This was contrary to our intuitive assumptions that the presence of multiple indications would increase the risk of failure of primary fascial closure. This should be explored further.

We found that patients with a logistical reason were most likely to have primary fascial closure compared to those without a logistical reason. Logistical determinants are frequently based upon a deliberate decision to return to the operating room rather than the status of the abdominal wall or the patient’s clinical condition. In logistical reasons, fascial closure is usually possible and would be physiologically tolerated but the decision is made not to close the abdomen. Several studies have demonstrated that patients who require fewer re-laparotomies, and who have shorter duration of open abdomen management have higher rates of primary fascial closure [25, 26]. Earlier closure has also been associated with improved post-operative outcomes [27]. In our series, logistical reasons, as expected, were associated with significantly higher rates of the bowel left in discontinuity and packing compared to the absence of that reason. In both of these clinical scenarios, a high rate of successful primary fascial closure would be expected at the first re-laparotomy [25]. The greater potential for obtaining early primary fascial closure is a defining characteristic of patients with logistical reasons and likely contributes to the high rate of fascial closure observed in this cohort.

Recently, Chiara et al. led an expert panel on open abdomens in trauma and published consensus recommendations defining the indications for its appropriate use [28]. The authors recommend the use of the open abdomen technique in several clinical scenarios, all of which can be organized into anatomical, logistical, and/or physiological categories described in our classification system. In that study, extreme visceral edema, retroperitoneal swelling, and elevated bladder pressure when closing harmonize with anatomical reasons for the open abdomen described herein. Logistical reasons include planned re-laparotomy for vascular/gastrointestinal injuries or mesenteric ischemia, packing, hematoma requiring a second look, and peritoneal contamination that has not resolved at the conclusion of the first operation [27]. Physiological reasons include, critically ill patients requiring abbreviated “damage control” procedures [28]. Similarly, the Eastern Association for the Surgery of Trauma Practice Management Guidelines recommends the use of the open abdomen in the context of severe abdominal trauma with intra-abdominal packing, acidosis, hypothermia, clinical coagulopathy, and massive transfusion [4]. Moreover, severe necrotizing pancreatitis and severe intrabdominal infection/peritonitis are part of the indications for the open abdomen in emergency general surgery [4]. Essentially, the three categories described in our study act as umbrella terms that encompass a broad range of clinical scenarios that could guide decision-making when considering the use of the open abdomen strategy. Therefore, we suggest that perhaps one should ask if there are any anatomical, logistical and or physiological reasons to leave the abdomen open before opting for this strategy. We consider that if none of these reasons are present the abdomen should be closed.

While the use of the open abdomen in trauma is widely endorsed by important guidelines, its use in the non-trauma setting is often contradictory [25]. In a recent meta-analysis of the open abdomen in non-trauma patients the most frequent indication was a planned re-laparotomy (or logistical according to our classification) [25]. This is in contrast to a retrospective review on damage control surgery in non-trauma patients where physiological reasons were more frequently described for leaving the abdomen open [27]. Moreover, practice patterns differ considerably in the non-trauma setting with some endorsing the use of the open abdomen only when the abdominal cavity cannot be physically closed [25]. The majority of our non-trauma patients had multiple indications for the open abdomen with both physiological and logistical reasons being present in all but one patient. Thus, our classification is conceptually useful for organizing the indications of the open abdomen in both the trauma and the non-trauma settings.

There are important limitations to our study. The majority of patients in our cohort had multiple reasons for undergoing an open abdomen procedure. This frequently resulted in the same patient being analyzed in more than one group. This potentially limits our ability to attribute with certainty specific clinical characteristics and outcomes to a single reason. In retrospect, it would have been ideal to have the surgeons in our study indicate their primary reason for using the open abdomen followed by their secondary and tertiary reasons when present. Analyzing patients into seven groups that would encompass all of the possible combinations of the three reasons would allow for more specific comparisons. Due to the relatively small sample size in each of these seven groups we were unable to obtain any meaningful analysis with such an approach in the present study. Since the primary intent of this study was to introduce a new classification system, patients were censored early during their hospital course limiting conclusions with respect to complications and long-term follow-up. The consequences of the limited use of negative pressure wound therapy in our cohort also apply to that limitation. We also observed a high mortality rate in our study interfering with a comprehensive analysis of the primary fascial closure rate in each category. Lastly, this study was conducted at a single-center with recruitment being limited to daytime hours; a multi-center trial with the potential for 24-h recruitment would improve patient accrual and provide means to further investigate the applicability of our classification.

## Conclusions

The classification, presented herein, proposes that all the indications for the open abdomen can be organized into three categories: anatomical; physiological; and logistical. These categories establish a practical and comprehensive terminology that could help to promote appropriate use of the open abdomen. Our findings suggest that there are significant differences among each of these categories with respect to clinical characteristics and primary fascial closure rates. Efforts are currently underway to conduct a large multi-center prospective observational study to validate this proposal.

## Abbreviations

ICU, intensive care unit; ISS, injury severity score; SBP, Systolic Blood Pressure; OR, odds ratio; CI, confidence interval
